# An Assessment of Usual Salt Intake among Older Normotensive Adults in Atonsu, a Suburb of Kumasi in the Ashanti Region of Ghana

**DOI:** 10.1155/2020/7053654

**Published:** 2020-10-21

**Authors:** Rufai Safianu, Jacob Plange-Rhule

**Affiliations:** ^1^Department of Basic Medical Sciences, University of Health and Allied Sciences, Ho, Ghana; ^2^Department of Physiology, Kwame Nkrumah University of Science and Technology, Kumasi, Ghana

## Abstract

**Background:**

Globally, sodium intake has been found to be far above the normal level required by the body. Within countries, variations in salt intake exist between rural communities and urban communities. Experimental and epidemiological studies as well as studies involving clinical trials show the existence of adverse effect of salt consumption on the blood pressure of adults. The study evaluated salt intake among older normotensive adults in Atonsu, a suburb of Kumasi in the Ashanti region of Ghana.

**Methods:**

Participants were randomly selected from five churches which constituted cluster samples. A questionnaire was administered to participants for demographic information and dietary and lifestyle assessments. The study targeted 100 participants, twenty from each of the five churches. Eighty-two individuals gave their informed consent. Out of the 82 who gave their informed consent, 15 withdrew and 67 completed the course. The 67 participants comprised 36 (53.7%) men and 31 (46.3%) women. Systolic and diastolic blood pressure, BMI, urinary sodium, urinary potassium, serum creatinine, serum sodium, and serum potassium concentrations were also measured.

**Results:**

Participants' mean age was 52.3 ± 8.7 years. Participants had 24 hr urinary sodium excretion of 153.0 ± 26.9 mmol/day. All participants indicated that they consume foods high in salt even though none of them added salt to their diet at table. Mean 24 hr urinary potassium was 52.5 ± 12.9 mmol/day. Mean systolic blood pressure was 119.9 ± 10.8 mmHg and mean diastolic blood pressure was 72.5 ± 7.3 mmHg. Their mean BMI was 23.7 ± 3.5 kg/m^2^.

**Conclusion:**

The participants who can be described as quite old and normotensive were high salt consumers, indicated by their dietary assessment and urinary sodium excretion, even though they had normal blood pressure.

## 1. Introduction

Experimental and epidemiological studies as well as studies involving clinical trials show the existence of adverse effect of salt consumption on the blood pressure of adults [1–3]. However, some individuals tend to be more salt-sensitive than others. Factors affecting salt sensitivity include age, race, and level of activity among others [4]. It has been demonstrated in Ghana that a long-term reduction in salt intake, by way of a long-term intervention programme, has the tendency of reducing blood pressure significantly [5]. Other studies have also shown the importance of dietary salt reduction in reducing not only the incidence of hypertension, but various cardiovascular diseases independent of hypertension [2, 6, 7].

The variations in salt (NaCl) consumptions of different populations around the world were first brought to the attention of the research community by a publication of Louis Dahl's famous graph in 1960 due to scientific revelations of its positive correlation with blood pressure [8]. Globally, sodium intake has been found to be far above the normal level required by the body [9]. A collaborative report by WHO/FAO on “Diet, Nutrition and the Prevention of Chronic Diseases” indicated that adults should take <85 mmol/day (2 g/day) of salt [9]. It has however been shown that adult salt consumption is in excess of 100 mmol/day (2.30 g/day) [9]. There are variations in salt intake between developed countries and developing countries [10]. Within countries, variations also exist between rural communities and urban communities. Within a population, variations exist between individuals [10].

Salt is widely consumed in Ghana by both rural and urban folks [5]. It is added to food in cooking, at table, and through a wide range of food additives and processed foods that are consumed at both home and public places knowingly or unknowingly. Among the major sources of salt in Ghana are all salted fish such as momoni and kako, and meat preserved with salt, such as beef and pig feet [5]. Cappuccio et al. studied the effects of dietary salt on blood pressure in rural and semi-urban communities in the Ashanti region of Ghana [5]. The study showed that urinary sodium excretion (*U*_NA_), an indication of salt intake, was comparable (99 vs 103 mmol/day) between rural and semi-urban communities. Rural communities, however, excreted more potassium than semi-urban communities [5]. Sodium is available in foods naturally, though in minute amounts. More sodium in the form of sodium chloride is also added to processed foods and home-cooked foods [11].

The major factors that motivate the addition of salt to foods are improvement in taste and flavour and not for health reasons [5].

Sodium is useful in human physiology as it is important in the establishment of fluid balance in the body, regulation of oncotic pressure, and neuromuscular physiology among others [12]. Nevertheless, studies show evidence of direct relationship between salt intake and incidence as well as prognosis of hypertension and stroke [3].

The prevalence of hypertension in Ghana has varied widely but has generally been higher in urban than in rural communities [13]. Rural communities consume less processed foods compared to urban communities [13]. It is necessary to evaluate salt intake of urban folks in Ghana since new packaged foods with “hidden salt” are added to existing ones on the market from time to time. It is important to assess salt intake in normotensive older adults since they are more susceptible to hypertension than younger people. The assessment would help in determining whether or not they are mindful of their salt intake and whether or not their normal blood pressure is partly as a result of low salt intake.

This study is a survey on normotensive adults in Atonsu, a suburb of Kumasi. Kumasi is a typical urban setting, being the second largest city in Ghana [14].

## 2. Methods

### 2.1. Study Design, Site, and Participants

The study has a cross-sectional design. It involves a survey of men and women forty years and above with normal blood pressure. The study was carried out at the Kumasi South Regional Hospital in the Ashanti Region of Ghana; this hospital is about a fifteen-minute drive from Kwame Nkrumah University of Science and Technology. The hospital's catchment area is made up of 56 communities, with approximately 400,000 people. The following clinical services are available in the hospital: pharmacy, surgical, general medicine, maternity, X-ray, regional reference laboratory, ultrasound, dental, optometry, and HIV Unit; as well as nonclinical services. The hospital offers 24-hour services on daily basis. Having adequate staff, it operates a 3-shift schedule.

### 2.2. Study Population

The population for the study was made up of healthy adult (40 years and older) residents (men and women), of Atonsu, a small suburb of Kumasi, who had normal blood pressure.

### 2.3. Sample

One hundred potential participants were selected as follows. Cluster samples of twenty people were selected from five churches each through medical screening exercise organized by the researcher in Atonsu, with the help of some health professionals. Names and telephone numbers of the screened people were recorded. Candidates meeting the inclusion criteria were chosen and out of these a sample of twenty people was randomly selected from each church to make a total of 100. In the selection process, individuals were identified with unique numbers. The numbers were written on pieces of paper and folded. A selection without replacement was done until the required number was attained. After explaining the study to the potential participants, their consent was sought. Eighty-two individuals gave their consent to participate. The participants filled consent forms that were approved by the Kwame Nkrumah University of Science and Technology Ethics Committee. Out of the 82 participants, made up of 42 females and 40 males, 67 (33 males and 27 females) completed the study.

### 2.4. Inclusion Criteria

The inclusion criteria included normotensive adults (males and females, 40 years and older) who were mentally sound and lived in Atonsu and who provided their consent.

### 2.5. Exclusion Criteria

The exclusion criteria included pregnant women, hypertensive individuals, people who drink alcohol habitually, and people with other chronic diseases such as diabetes.

### 2.6. Data Collection Procedure

Data collection was carried out from 2^nd^ August, 2015, to 5^th^ October, 2015. After filling the consent form, a questionnaire was administered to each participant to seek demographic, dietary, and lifestyle information. 5 ml of blood samples was taken for the measurement of serum sodium, potassium, and creatinine concentrations. Participants were instructed to submit their two 24 hr urine samples, thus on the 3^rd^ and 4^th^ days. The urine volumes were measured with measuring cylinder and small samples of the urine were then taken for the measurement of urinary sodium and potassium concentrations. Blood pressure, weight, and height were also measured.

#### 2.6.1. Instructions to Participants and 24 hr Urine Sample Collection

Participants were given clean plastic bottles for urine collection. In addition to the bottles, female participants were given funnels. They were asked to void their first morning urine and note the time. All subsequent urine samples were to be collected into the bottle till the same time the next day [15]. They were asked to keep the urine in a cool environment, if possible, in the fridge. The samples were then submitted to the researcher at the hospital. They were then given new bottles to start the next 24 hr collection and submit another sample the next day following the same protocol. 24 hr urine volume was measured with a measuring cylinder and shaken well. 8 ml sample was then taken into a plastic tube and frozen at −80°C.

#### 2.6.2. Collection and Preservation of Venous Blood

New sterile 5 ml syringes with needles were used to take 5 ml peripheral whole blood sample from each participant into well-labeled sterile test tubes (yellow top vacutainers) manufactured by Narang Medical Ltd, China. The blood was spun at 4000 rpm for 10 minutes. The serum was transferred into a well-labeled sterile Eppendorf tube and stored at −80°C.

#### 2.6.3. Serum and Urine Biochemistry Analyses

Selectra Junior autoanalyzer, manufactured by the ELITech group in USA, was used to measure the concentrations of serum sodium, serum potassium, and serum creatinine.

#### 2.6.4. Urine Sodium and Urine Potassium

Concentrations of urine analytes were multiplied by their respective 24 hr urine volumes to achieve 24 hr concentrations of the analytes. The machine was calibrated for each analyte prior to analysis.

#### 2.6.5. Serum Sodium Concentration

The reagent for the measuring sodium concentration contains selective chromogen, which reacts with sodium to produce a chromophore whose absorbance varies directly with the concentration of sodium in the test specimen. The sodium standard contained 150 mm/L sodium. 10 *μ*L of the sample reacted with 1000 *μ*L of the reagent with incubation period of 5 min at room temperature. Absorbance was measured against reagent blank at 630 nm wavelength.

The spectrophotometer was calibrated to compute the sample concentration as follows:

(1) $$\begin{array}{c} \frac{\text{AT}}{\text{AS}}\times \text{concentration of standard,} \end{array}$$

where AT = absorbance of sample and AS = absorbance of standard.

#### 2.6.6. Potassium Concentration

The reagent for measuring potassium concentration contains sodium tetraphenylboron, which reacts with potassium to produce a turbid dispersed suspension of potassium tetraphenylboron. The turbidity of the suspension is proportional to the concentration of potassium. The potassium standard contained 5.0 mmol/L potassium. 20 *μ*L of the sample was mixed with 1 ml and 20 *μ*L of distilled water for incubation period of 5 minutes at 630 nm wavelength. The machine was calibrated to compute the sample concentration as follows:

(2) $$\begin{array}{c} \frac{\text{AT}}{\text{AS}}\times \text{concentration of standard,} \end{array}$$

where AT  =  absorbance of sample and AS = absorbance of standard.

#### 2.6.7. Creatinine Concentration

Two reagents, *R*_1_ and *R*_2_, were used for determining creatinine concentration. *R*_1_ contained picric acid (8.73 mmol/L). *R*_2_ composed of sodium hydroxide (312.5 mmol/L) and disodium phosphate (12.5 mmol/L). One volume of *R*_1_ was mixed with one volume of *R*_2_. A standard solution with concentration 2 mg/dL was also used. The rate of formation of coloured complex between creatinine and alkaline picrate was measured. The effects of interfering substances were reduced using the kinetic procedure. Concentrations were measured against distilled water at the wavelength of 500 nm at room temperature. The machine was calibrated to compute the sample concentration as follows:

(3) $$\begin{array}{c} \left(\text{A}2-\text{A}1\right)\text{sample}\times n,\\ \left(\text{A}2-\text{A}1\right)\text{standard}, \end{array}$$

where A1 = first absorbance, A2 = absorbance after 2 minutes, and *n* = standard concentration (2 mg/dL).

To confirm the reliability of the protocol, the researcher performed five serial dilutions of the standard solution and plotted a linear graph of concentration against absorbance. A correlation coefficient of 0.98 was obtained. The protocol was therefore reliable.

#### 2.6.8. Urine Analysis

Urine sodium and potassium levels of 24 hr urine were determined. Urinary levels of sodium and potassium were quantified using an indirect potentiometer with selective solid membranes for each ion connected to an AU 5400 Autoanalyzer (Olympus, Mishima, Japan): coefficient of variation (CV) = 1.0% for sodium and 1.1% for potassium.

#### 2.6.9. BMI Determination

To determine BMI, weight and height were measured using standard protocols given by Weiner and Lourie [16]. Weight was measured with electronic balance scale (Seca 875). In measuring the weight, participants were told to remove their shoes and rid their body of other items except their dress. Stadiometer was used to measure height. Participants were instructed to stand erect on the stadiometer without wearing shoes. To calculate body mass index (BMI), the weight in kilograms was divided by the square of the height in meters (kg/m^2^) from the formula BMI = weight (kg)/height (m^2^) [17].

#### 2.6.10. Blood Pressure Measurement

Systolic and diastolic pressure of participants were measured on the right arm with a digital sphygmomanometer (Omron 907XL) three times at 30-second intervals and the averages were recorded. Participants were rested in a sitting position for five minutes before the blood pressure was measured. All pressure measurements were done in the morning and the time was recorded [18].

#### 2.6.11. Data Handling and Analysis

The questionnaires and completed laboratory forms were kept in a safe locker. The data was subsequently entered into Microsoft Excel and analyzed with Statistical Package for Social Sciences (SPSS), version 16.0. Mean values were determined by way of descriptive statistics. Unpaired *t*-test for sex groups and ANOVA (analysis of variance) for BMI and age groups were carried out to compare means at 0.05 significance level in subgroup analyses. Data was protected with a password.

## 3. Results

### 3.1. Demographics and Lifestyle Information

**Figure 1:**
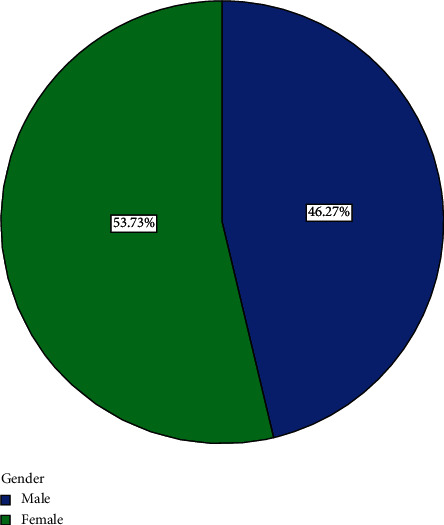
Sex distribution.

**Figure 2:**
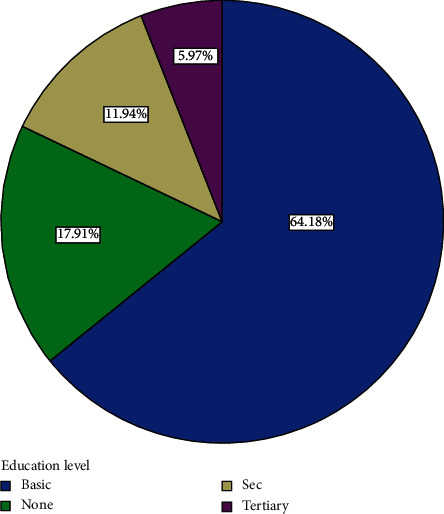
Distribution of educational level of participants.

**Figure 3:**
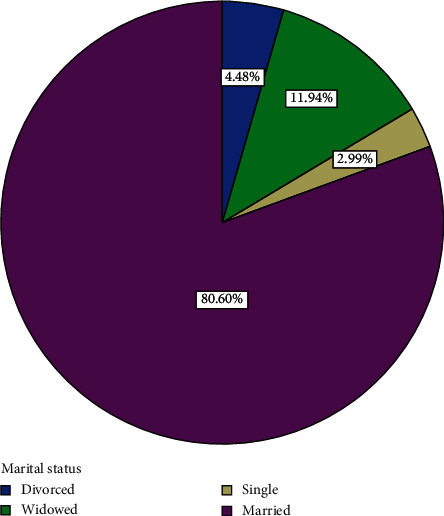
Distribution of marital status of participants.

**Figure 4:**
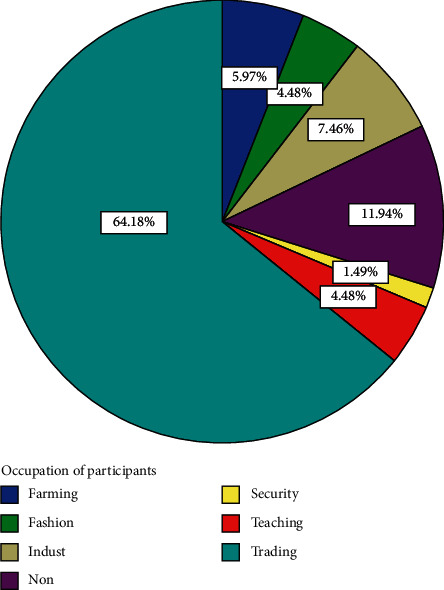
Distribution of occupation of participants.

**Figure 5:**
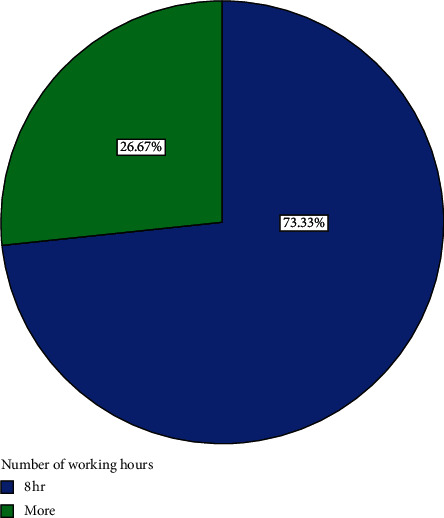
Distribution of number of hours participants worked.

**Figure 6:**
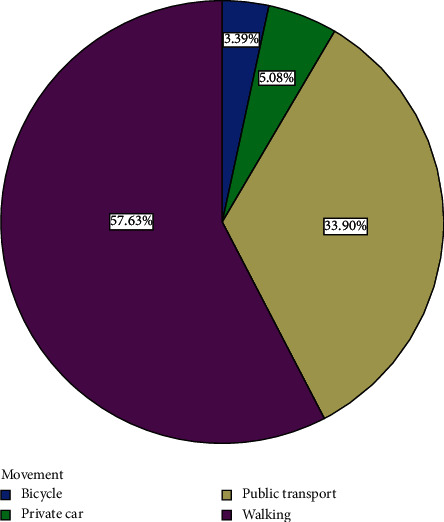
Distribution of means of transportation of participants to their workplaces.

Out of the 82 participants recruited for the study, 15 withdrew and 67 completed the course. Analyses are therefore based on the 67 that completed the course. The 67 participants comprised 36 (53.73%) men and 31 (46.27%) women (Figure 1). Forty-three, representing 64.2% of participants, had basic education (Figure 2). Eight, representing 11.9% of participants, had secondary education (Figure 2). Four (6%) had tertiary education while 12 (17.9%) had none (Figure 2). Fifty-four (80.60%) were married, three (4.48%) were divorced, and eight (11.94%) were widowed while 2 (2.99%) were single (Figure 3). Forty-three (64.2%) of the participants were traders (Figure 4). Four (6.0%) were farmers (Figure 4). Three (4.5%) were into fashion design (Figure 4). Five (7.5%) were engaged in industrial work (Figure 4). One (1.5%) was a security person while 3 (4.5%) were teachers (Figure 4). Eight (11.9%) of the participants indicated that they were unemployed (Figure 4). Out of those who were employed, 74.2% usually worked 8 hours or less a day while 25.8% worked for more than eight hours a day (Figure 5). Fifty-seven (63%) of those who were employed usually walked to work (Figure 6). 33.90% usually went to work by public transport (Figure 6). 3.39% indicated that they went to work using bicycle while 5.08% usually went to work using their private cars (Figure 6).

**Figure 7:**
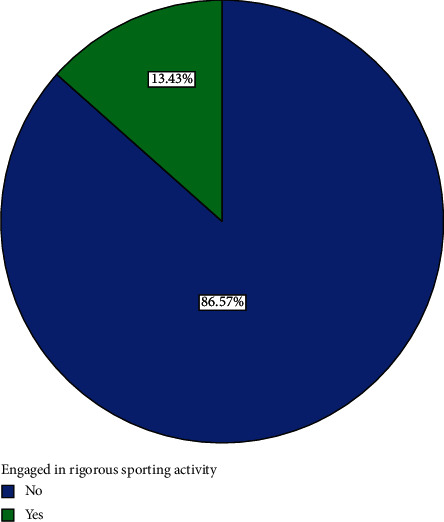
Distribution of whether or not participants engaged in rigorous sporting.

Nine (13.4%) participants engaged in rigorous sporting activities while 58 (86.60%) did not do any rigorous sporting activities (Figure 7).

**Figure 8:**
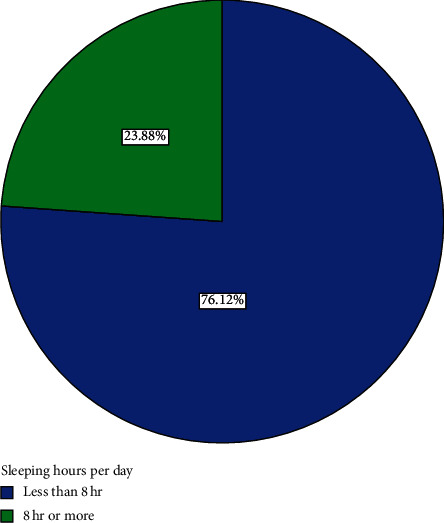
Distribution of sleeping hours per day of participants.

**Figure 9:**
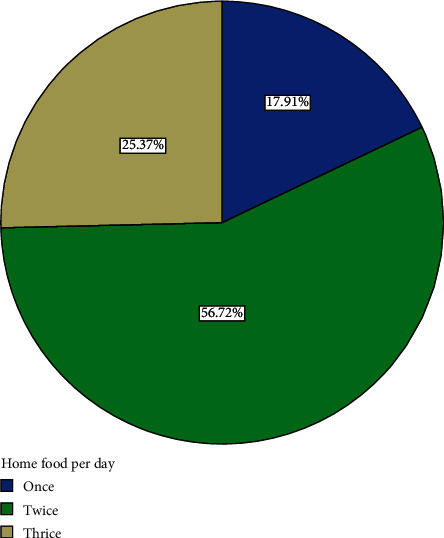
Distribution of number of times participants ate home-cooked food per day.

**Figure 10:**
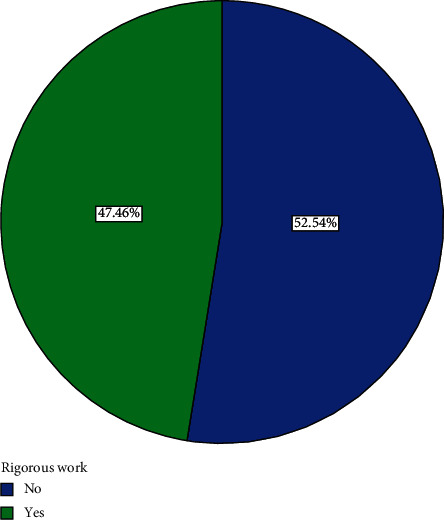
Distribution of whether participants' work involved rigorous activities.

Sixteen (23.9%) participants indicated that they usually slept for 8 hours or more a day while 51 (76.1%) usually slept for less than 8 hours a day (Figure 8). Fifty-six (72%) of the participants usually ate home-cooked food twice a day (Figure 9). 25.37% usually ate home-cooked food thrice a day while 17.91% ate home-cooked food once a day (Figure 9). All participants (100%) had salt added to their diets in cooking, though none of the participants added salt to their diet at table. 47.46% of those who were employed did rigorous activities in their work while 52.54% did not do rigorous activities in their work (Figure 10).

### 3.2. Mean Values of Continuous Variables

The mean variables are presented as mean ± SD in Table 1. The mean age of participants was 52.3 ± 8.7. Regarding the biochemistry variables, 24 hr urine sodium was 153.0 ± 26.9 mmol/L, 24 hr urinary potassium was 52.5 ± 12.9 mmol/L, serum sodium was 150.1 ± 7.1, serum potassium was 3.5 ± 0.6 mmol/L, and serum creatinine was 1.0 ± 0.1 mg/dL. Regarding blood pressure, the mean systolic and diastolic pressure were 119.9 ± 10.8 mmHg and 72.5 ± 7.3 mmHg, respectively. The mean BMI was 23.7 ± 3.5 kg/m^3^.

### 3.3. Stratification of Urinary Sodium Excretion by Sex, Age, and BMI

The stratification of urinary sodium and potassium excretion is presented in Tables 2 and 3. There was no significant difference in 24 hr urinary sodium excretion between males and female (*p*=0.73). After putting the ages of participants into three groups, there was no association between age group and 24 hr urinary sodium excretion (*p* value = 0.35). BMI was also not associated with 24 hr urinary sodium (0.96).

## 4. Discussion

Characteristics of participants were evaluated in line with the objectives of the current study. The results evaluated usual salt intake and systolic and diastolic blood pressure as well as potential confounding factors such as age, lifestyle, diet, and BMI of participants. As approximately 90% of sodium consumed is excreted via urine, an accurate 24 hr urine collection, as was done in the current study, reflects intake reliably [15]. According to literature, variable losses in sodium also occur through sweat and faeces and have been estimated to be around 10% under normal conditions [15].

The mean usual sodium excretion (153.0 mmol/day), according to available literature, can be described as high and that it puts participants at a high risk of becoming hypertensive in the future [9]. However, their high potassium intake indicated by their 24 hr potassium excretion of 52.5 ± 12.9 mmol/day might reduce the effect of the high sodium [19].

Urine sodium and potassium excretions were also stratified by sex, age, and BMI. The results suggest that their sodium intake indicated by 24 hr urinary sodium excretion was not associated with sex (*p*=0.73), age (*p*=0.35), and BMI (*p*=0.99) (Table 2). However, it has recently been shown that high salt intake is associated with an increased risk of obesity through sugar-sweetened beverage consumption [20, 21]. Potassium intake, indicated by 24 hr urinary potassium excretion, was not associated with sex (*p*=0.82), age (*p*=0.40), and BMI (*p*=0.81) (Table 3).

The only study on sodium intake in Ghana, by Cappuccio et al., found the mean baseline sodium excretions to be 99 mmol/day and 103 mmol/day in rural and semi-urban settings, respectively [5]. This is at variance with the results in the current study. The current study was conducted in an urban setting. The difference in the results is a confirmation of the higher consumption of salt laden foods in urban than in rural and semi-urban communities [13], even though the current study targeted only normotensive healthy older adults. All participants in the current study indicated that, in addition to the salt laden packaged foods they consume, they also add salt to their foods in cooking, though none of them indicated that they added salt to food at table. The mean serum sodium, serum potassium, and serum creatinine concentrations of participants were normal, going by standard reference ranges [22]. Typically, plasma sodium concentration cannot be changed beyond 5% by the consumption of salt, no matter the quantity [23]. It is however used for diagnostic purposes [23].

With the average age of 52.3 years, the participants could be described as being old enough for the blood pressure effect of high salt intake to be anticipated. Older people are more sensitive to salt than younger people [4]. However, genetic variation may play a role in making some older people being less salt-sensitive than other people of their age. Blood pressure classifications in literature [24] support the fact that the mean systolic and diastolic blood pressures in the current study (119.9 and 72.5 mmHg, respectively) of the participants were normal [25], as they were below 130 mmHg and 85 mmHg, respectively.

Most participants had basic education (Figure 2). They were therefore expected to have basic knowledge of some of the health implications of dietary ingredients, in this case salt. Poor education has negative effect on a person's choice of diet. Moreover, the results on lifestyle suggest that most participants had enough sleep (Figure 8) and were not overworking since the majority of them (76.1%) did not work for more than eight hours a day even though a small minority were jobless. Their mean BMI was also normal. They can also be described as active people since, per the results, most of those who were working engaged in rigorous work, even though most of them were not involved in rigorous sporting activities. The majority, yet again, walked to their work places. Regular walking is a good form of exercise as supported by findings of various studies [26]. These are all contributory factors for their good health.

## 5. Conclusion

According to the 24 hr urinary sodium excretion and the dietary assessment with respect to salt intake, sodium, and for that matter salt intake, can be described as higher than the recommended amount. Reducing salt intake in older normotensive adults in an urban community needs to be targeted in efforts aimed at reducing the incidence of cardiovascular diseases.

## Figures and Tables

**Table 1:** Mean values of baseline characteristics.

Parameter	Mean	Std. dev.
Age (years)	52.3	8.7
24 hr urine sodium (mmol/day)	153.0	26.9
24 hr urine potassium (mmol/day)	52.5	12.9
Serum sodium (mmol/L)	150.1	7.1
Serum potassium (mmol/L)	3.5	0.6
Serum creatinine (mg/dL)	1.0	0.1
Systolic blood pressure (mmHg)	119.9	10.8
Diastolic blood pressure (mmHg)	72.5	7.3
BMI (kg/m^2^)	23.7	3.5

**Table 2:** 24 hr urinary sodium excretion (UNa) stratified by sex, age, and BMI.

Group	*N*	24 hr UNa (mmol/day)	Std dev	*p* value
(*Sex*)				
Male	31	154.2	27.3	0.73
Female	36	151.9	26.9

(*Age*)				
40–49	33	151.9	27.0	0.35
50–59	16	147.1	18.9
60 and above	18	160.2	32.1

(*BMI*)				
<18	7	151.4	26.9	0.99
18–25	28	153.3	28.2
>25	32	153.1	26.6

**Table 3:** 24 hr urinary potassium (UK) excretion stratified by sex, age, and BMI.

Group	*N*	24 hr UK (mmol/day)	Std dev	*p* value
(*Sex*)				
Male	31	52.1	12.5	0.82
Female	36	52.9	13.4	

(*Age*)				
40–49	33	50.4	13.9	0.40
50–59	16	54.3	11.2	
60 and above	18	54.9	12.4	

(*BMI*)				
<18	7	54.2	5.2	0.81
18–25	28	51.5	13.2	
>25	32	53.5	14.1	

## Data Availability

The data used to support the findings of this study are available from the corresponding author upon request.

## Abbreviations

ANOVA: Analysis of variance
BMI: Body mass index
HIV: Human immunodeficiency virus
SD: Standard deviation
SPSS: Statistical Package for Social Science
UK: Urinary potassium
UNa: Urinary sodium.
